# Anatomic vs. reverse shoulder arthroplasty for the treatment of Walch B2 glenoid morphology: a systematic review and meta-analysis

**DOI:** 10.1016/j.xrrt.2021.08.003

**Published:** 2021-09-01

**Authors:** G. Bradley Reahl, Hussein Abdul-Rassoul, Ryan L. Kim, Kyle S. Ardavanis, David Novikov, Emily J. Curry, Joseph W. Galvin, Josef K. Eichinger, Xinning Li

**Affiliations:** a Department of Orthopaedic Surgery, Boston University School of Medicine, Boston, MA, USA; b Department of Orthopaedic Surgery, Madigan Army Medical Center, Joint Base Lewis-McChord, WA, USA; c Medical University of South Carolina, Department of Orthopaedics, Charleston, SC, USA

**Keywords:** Walch B2 glenoid, Glenohumeral osteoarthritis, Total shoulder arthroplasty, Reverse shoulder arthroplasty, Eccentric reaming, Posteriorly augmented glenoid component, Systematic review

## Abstract

**Background:**

Walch B2 glenoid morphology with glenohumeral osteoarthritis is a difficult degenerative pattern to manage for shoulder surgeons. Anatomic total shoulder arthroplasty (TSA) in combination with eccentric reaming or bone grafting are the traditional methods of treatment. Newer approaches such as TSA with posteriorly augmented glenoid components and reverse shoulder arthroplasty (RSA) may offer better stability for the posteriorly subluxated biconcave B2 wear pattern. The aim of this systematic review is to compare mid-term surgical and functional outcomes of Walch B2 glenoids without significant rotator cuff pathology treated with TSA and RSA.

**Methods:**

The review was performed according to Preferred Reporting Items for Systematic Reviews and Meta-Analyses (PRISMA) guidelines by searching the MEDLINE (PubMed) and Embase (Elsevier) databases. Inclusion criteria were clinical studies that evaluated the outcomes and complications of TSA or RSA in the setting of B2 glenoid morphology without significant rotator cuff pathology. Data relevant to TSA and RSA surgical outcomes were extracted and compiled, and outcomes were compared. A meta-analysis of proportions of complication and revision rates among TSA and RSA groups was performed.

**Results:**

Overall, 16 articles were included with 414 TSAs and 78 RSAs. The average follow-up duration was 54.1 ± 14.8 months for patients undergoing TSA and 44.8 ± 10.1 months for patients undergoing RSA. The TSA group was further subdivided based on the use of eccentric reaming (135 TSAs), an augmented glenoid component (84 TSAs), or bone grafting (11 TSAs) or was unspecified (184 TSAs). Overall, patients undergoing TSA and RSA demonstrated mean improvements of 50.1 ± 8.5° and 64.7 ± 5.2° in active flexion, 58.5 ± 10.3° and 68.9 ± not reported° in active abduction, and 31.3 ± 5.7° and 29.0 ± 10.2° in active external rotation, respectively. In regard to functional outcome scores, patients undergoing TSA and RSA showed mean Constant score improvements of 38.8 ± 5.3 and 46.6 ± 3.1 points and American Shoulder and Elbow Surgeons score improvements of 48.2 ± 1.0 and 49.2 ± 25.3 points, respectively. Results of the meta-analysis with mid-term follow-up data demonstrated pooled complication rates of 9% (95% confidence interval [CI], 1%-22%) for TSA and 6% (95% CI, 0%-28%) for RSA and pooled revision rates of 2% (95% CI, 0%-8%) for TSA and 1% (95% CI, 0%-15%) for RSA.

**Conclusion:**

In the setting of Walch B2 glenoid morphology, TSA with eccentric reaming or an augmented component yields comparable outcomes to RSA. Based on the patient’s age, activity level, and expectations, both TSA and RSA can be considered a reasonable option to treat Walch B2 glenoid morphology.

Primary glenohumeral osteoarthritis is a painful and debilitating degenerative condition that can be treated with either anatomic total shoulder arthroplasty (TSA) or reverse total shoulder arthroplasty (RSA). Surgical indications, outcomes, and complications after shoulder arthroplasty are associated with both rotator cuff status and glenoid deformity. Of the glenoid deformities, the Walch B2 morphology is especially challenging to address.^6^^,^^9^^,^^11^^,^^12^^,^^17^, ^18^, ^19^, ^20^^,^^26^^,^^30^^,^^45^ Characterized principally by posterior glenoid erosion and posterior humeral head subluxation, B2 glenoids have a biconcave morphology with native paleo-glenoid and eroded neoglenoid surfaces.^22^^,^^44^^,^^45^ Static posterior humeral head subluxation contributes to the posterior glenoid erosion, particularly in patients with significant premorbid glenoid retroversion and/or a flat posterior acromial slope.^17^^,^^22^^,^^31^ In addition, posterior capsular laxity, anterior capsular contracture, and fatty infiltration of the rotator cuff musculature have all been associated with the B2 wear pattern.^7^^,^^12^

Controversy exists as to which arthroplasty technique best addresses the complexities created by the rotator cuff intact (RCI) B2 glenoid morphology. Traditionally, these deformities have been treated with anatomic TSA performed in conjunction with eccentric reaming (ER) to correct glenoid version.^18^^,^^26^^,^^30^ This technique is limited, however, as correction of >15° of retroversion results in subchondral bone removal and joint line medialization that can cause posterior inferior peg perforation precipitating glenoid component loosening (GCL) and decreased joint stability.^4^^,^^26^^,^^30^ Alternatively, TSA with bone grafting (BG) or the use of a posteriorly augmented glenoid component (PAGC) can permit version and subluxation correction without bone removal. However, the long-term clinical outcomes of these techniques are characterized by either inconsistent results or, in the case of PAGC, a lack of sufficient outcome data.^12^^,^^21^^,^^26^^,^^30^^,^^35^^,^^45^ Overall, the difficulty in addressing the severe biconcave posterior wear and posterior humeral head subluxation with TSA has translated into more frequent GCL, higher incidences of radiolucent lines, increased revision rates, and lower functional outcome scores than other glenohumeral arthritic patterns.^6^^,^^9^^,^^11^^,^^12^^,^^17^, ^18^, ^19^, ^20^^,^^26^^,^^30^^,^^45^

Although previously used only in patients with rotator cuff tear arthropathy, several authors have recently reported good to excellent midterm joint stability, improved functional outcome, and low rates of baseplate loosening using RSA for the B2 glenoid morphology with an intact rotator cuff.^6^^,^^17^^,^^24^^,^^25^^,^^29^^,^^32^ These findings are likely due to the semiconstrained design and robust glenoid baseplate fixation in RSA, eliminating complications created by posterior humeral head subluxation and decreasing the likelihood of GCL.^6^^,^^17^^,^^30^ Despite these results and advantages, little investigation has been performed directly comparing the use of RSA to TSA to treat B2 glenoid deformities, with most studies reporting outcomes of each procedure individually in limited sample sizes. The purpose of this systematic review was to assess range of motion (ROM), functional outcomes, revision rates, and complications of B2 glenoids without significant rotator cuff pathology treated with RSA in comparison to TSA.

## Materials and methods

### Search strategy

**Figure 1:**
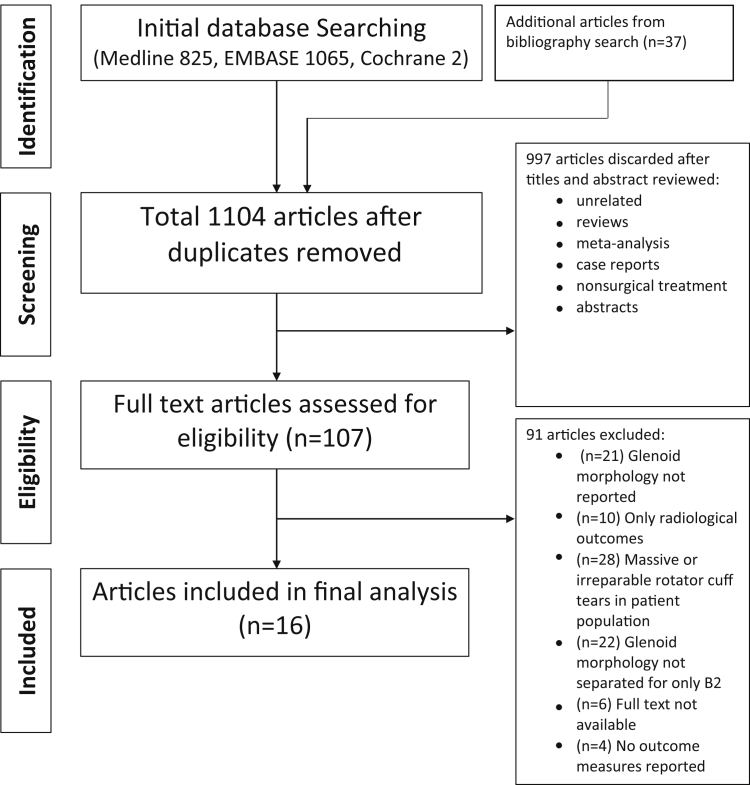
PRISMA (Preferred Reporting Items for Systematic Meta-Analyses) flow chart detailing selection process of included studies for final analysis.

A systematic and rigorous search strategy was developed according to the Preferred Reporting Items for Systematic Reviews and Meta-Analyses (PRISMA) guidelines (Fig. 1).^33^^,^^43^ This strategy yielded appropriate peer-reviewed data and articles for a systematic review over 4 phases. In phase 1, “identification,” electronic databases were searched to find relevant TSA and RSA articles. Medline (PubMed), Embase (Elsevier), and the Cochrane Library were accessed and searched on April 19, 2020, with the following Boolean search terms ((“Shoulder arthritis”) or (“Glenohumeral arthritis”) or (“Shoulder Osteoarthritis”) or (“Glenohumeral osteoarthritis”) or (“Glenoid erosion”) or (“Glenohumeral erosion”) or (“glenoid biconcavity”) or (“Biconcave glenoid”) or (“B2 glenoid”) or (“Walch B2 Glenoid”) or (“Glenoid retroversion”) or (“B2 Deformity”) or (“glenoid wear”)) AND ((“Total Shoulder Replacement”) or (“Reverse Shoulder Replacement”) or (“Total Shoulder Arthroplasty”) or (“Reverse Shoulder Arthroplasty”) or (“Ream and Run”) or (“augmented Glenoid”) or (Arthroplasty) or (“Glenoid reaming”) or (“Shoulder replacement”)).

### Eligibility criteria

All search returns were extracted and examined for relevance, and duplicate search returns were discarded. In phase 2, “screening,” titles and abstracts were screened for relevance. Bibliographies of relevant articles were also manually searched for other relevant articles screened out of the database algorithms. Articles were filtered out per the following exclusion criteria: (1) non-English text, (2) revisional cases, (3) only abstract available, (4) hemiarthroplasty only, (5) glenoid morphology other than biconcave (B2) glenoid (6) significant concomitant rotator cuff pathology, (7) review article or meta-analysis, or (8) case reports. Of note, an exception to one of these criteria was made for the included article by Magosch et al^27^ which had German text that could be reliably translated using a Google translation plugin. As the article passed all other phases of review, the authors deemed it appropriate for final inclusion. The shoulder osteoarthritis treatments being reviewed included TSA and RSA regarding outcomes specifically pertaining to the Walch classification B2 glenoid. Studies were evaluated only if they were (1) primary TSA or primary RSA; (2) included glenoids with biconcave morphology (Walch B2); (3) included postoperative outcomes such as American Shoulder and Elbow Surgeons (ASES) score, Constant score, ROM, and complications; and (4) reported a minimum of 1 year of follow-up (FU).

### Article review

In phase 3, “eligibility,” all articles eligible after the screening phase were evaluated for inclusion criteria and relevant data on outcomes after 1 of the surgical treatments of interest. All articles were reviewed, assessed, and data-mined by 3 independent evaluators. All results were then compared to ensure consistency and accuracy. Any conflicts or issues were resolved by review, and in the event of further disagreement, the final decision was made by the senior author (X.L.).

### Data extraction and assessment

In phase 4, “included,” articles that met inclusion criteria were analyzed for quality, and data were extracted to be used in a meta-analysis. The following items of data were extracted from the included articles: author; publication year; journal title; level of evidence; study design; surgical procedure; number of patients; sex ratio (M:F); mean age at the time of surgery; glenosphere position; concomitant procedures; glenoid correction technique including ER, BG, and PAGC; FU time; active preoperative ROM and postoperative ROM at the final FU in flexion, abduction, and external rotation; preoperative Constant score; postoperative Constant score at the final FU; preoperative ASES score; postoperative ASES score at the final FU; complications; and subsequent procedures.

### Quality assessment

To assess the quality of each case series that was included in the analysis, the Risk of Bias in Non-Randomized Studies of Interventions (ROBINS-I) tool is typically applied.^41^ However, most studies included in this analysis were of level III or IV evidence, and therefore, applying the ROBINS-I criteria was unnecessary.

### Statistical analysis

A meta-analysis of proportions of complication and revision rates among RSA and TSA was performed as this was the most consistent outcome variable that was amenable to this type of analysis reported. The meta-analysis was conducted by using a Freeman-Turkey transformation (arcsine square root transformation) under the random-effects model to calculate pooled estimate rates, whereas 95% confidence intervals (CIs) were estimated with the DerSimonian-Laird estimator.^10^ In an effort to account for differences among studies in regard to patient characteristics, surgical characteristics, and study methodology, the random-effects model was chosen.^3^ The heterogeneity analysis of the included studies was represented using I^2^, where I^2^ represents an estimated percentage of error attributed to interstudy variation.^15^ Based on the Cochrane review handbook, values of I^2^ between 0 and 40% were deemed to not be important, 30%-60% represented moderate heterogeneity, 50%-90% substantial heterogeneity, and 75%-100% considerable heterogeneity.^14^ Data were collected and stored in Microsoft Excel (Microsoft Corporation, Redmond, WA) and were further analyzed using R version 3.41 (R Foundation for Statistical Computing, Vienna, Austria) with the metaphor package.^42^

## Results

After a careful search and review of the currently available literature, 1 level II,^13^ 7 level III,^2^^,^^8^^,^^23^^,^^27^^,^^38^^,^^46^^,^^47^ and 8 level IV^5^^,^^12^^,^^21^^,^^28^^,^^32^^,^^36^^,^^39^^,^^45^ studies were ultimately included. These studies included those that assessed RSA and TSA for shoulders without significant rotator cuff pathology. Of these, 11 studies^8^^,^^12^^,^^13^^,^^21^^,^^23^^,^^28^^,^^36^^,^^38^^,^^39^^,^^45^^,^^47^ assessed TSA exclusively, 3 studies^5^^,^^32^^,^^46^ assessed RSA exclusively, and 2 studies^2^^,^^27^ assessed both TSA and RSA. A total of 78 shoulders underwent RSA and 414 shoulders underwent TSA. Of those that received a TSA, 135 shoulders underwent ER, 11 shoulders received BG, and 84 shoulders received a PAGC. For the remaining 184 shoulders that received a TSA, the glenoid correction technique was not specified. One-hundred sixteen of these 184 shoulders were from the articles by Walch et al^45^ and Habermeyer et al^13^ in which the authors describe select patients who received BG in addition to ER but did not uniquely stratify out the results for these patients. Therefore, we could not reliably include them in either the TSA + ER or TSA + BG subgroups. In total, there were 4 TSA subgroups analyzed: (1) TSA + ER, (2) TSA + PAGC, (3) TSA + BG, and (4) TSA + unspecified glenoid approach (UGA). Among the demographic data that could be extracted, the average age of patients undergoing TSA was 63.7 ± 1.8 years vs. 72.8 ± 1.3 years for patients undergoing RSA. In addition, the average FU was 54.1 ± 14.8 months for patients undergoing TSA and 44.8 ± 10.1 months for patients undergoing RSA. Complete demographics provided by each study are detailed in Table I for patients undergoing TSA and in Table II for patients undergoing RSA and compared as weighted averages in Table III.

**Table I:** Demographic data for included TSA studies.

First author & year	Level of evidence	Glenoid correction technique	Patients (shoulders), n	Sex ratio, M:F	Mean age at surgery (range or SD), years	Mean follow-up time (range or SD), months	Concomitant procedures/notes
Alentorn-Geli et al, 2018^2^^,^∗	III	ER	15 (15)	14:1	70.5 (±7.5)	42.7 (±18.4)	None
Egger et al, 2019^8^	III	ER	15 (15)	20:4†	59.0 (±7.2)†	42.6 (24-74)†	None
Leschinger et al, 2017^23^	IV	ER	27 (27)	78:25†	66.0 (37-83)†	77.6 (36-140)†	12 patients of unspecified glenoid morphology had small rotator cuff defects (<1 cm with stable joint control) which were evaluated and repaired intraoperatively
Magosch et al, 2017^27^	IV	ER	15 (15)	28:20†	67.3 (46-77)†	49.0 (24-77)†	None
Orvets et al, 2018^36^	IV	ER	59 (59)	36:23	64.0 (38-84)	50.0 (24-97)	None
Pastor et al, 2015^37^	IV	ER	4 (4)	20:21†	68.4 (56-78)†	22.4 (12-57)†	None
Grey et al, 2020^12^	IV	PAGC	46 (46)	30:16	65.1 (±8.0)	51.9 (±18.3)	None
Rice et al, 2008^38^	IV	PAGC	13 (14)	12:1	66.0 (52-78)	60.0 (25-96)	None
Wright et al, 2015^47^	IV	PAGC	24 (24)	17:7	65.8 (±11.5)	29.4 (±7.9)	None
Klika et al, 2014^21^	IV	BG	11 (11)	5:6	64.6 (±7.3)	83.7 (±84.4)	None
Habermeyer et al, 2007^13^	IV	Unspecified	24 (24)	30:47†	67.6 (47-85)†	24 (12-84)†	None
Magosch et al, 2017^27^^,^∗	IV	Unspecified	68 (68)	39:29	64.9 (±9.7)	48.6 (±27.6)	None
Walch et al, 2012^45^	IV	Unspecified	75 (92)	29:46	68.0 (50-85)	77.0 (14-180)	9 shoulders received a posterior capsulorrhaphy due to intraoperative instability in neutral rotation; 6 shoulders received a humeral head osteotomy with a decreased humeral retroversion when the planned glenoid retroversion on preoperative CT was between 8° and 10°.

*TSA*, total shoulder arthroplasty; *SD*, standard deviation; *ER*, eccentric reaming; *PAGC*, posteriorly augmented glenoid component; *BG*, bone grafting; *RSA*, reverse shoulder arthroplasty.

∗ Study includes both RSA and TSA B2 patients.

† Not stratified for B2’s, total sample data reported.

**Table II:** Demographic data for included RSA studies.

First author & year	Level of evidence	Patients (shoulders), n	Sex ratio, M:F	Mean age at surgery (range or SD), years	Mean follow-up time (range or SD), months	Glenosphere size, mm	Bone grafting, yes/no (patients)	Concomitant procedures/notes
Alentorn-Geli et al, 2018^2^^,^∗	III	16 (16)	11:5	72.5 (±5.4)	35.1 (±14.2)	36 or 41	Yes (4)	None
Collin et al, 2019^5^	IV	15 (15)	6:9	72.0 (NR)	92.4 (60-192)†	NR	Yes (NR)	None
Magosch et al, 2017^27^^,^∗	IV	7 (7)	3:4	70.1 (±6.7)	31.8 (±15)	NR	NR	NR
Mizuno et al, 2013^32^	IV	27 (27)	5:22	74.1 (66-82)	54.0 (24-139)	36 or 42	Yes (10)	6 patients had procedures on the contralateral shoulder: 2 TSA, 2 HA, 2 RSA
Waterman et al, 2020^46^	III	13 (13)	20:23†	74.7 (±7.1)†	32.3 (±12.3)†	NR	Yes (NR)	NR

*RSA*, reverse shoulder arthroplasty; *SD*, standard deviation; *NR*, not reported; *TSA*, total shoulder arthroplasty; *HA*, hemiarthroplasty.

∗ Study includes both RSA and TSA B2 patients.

† Not stratified for B2’s, total sample data reported.

**Table III:** Comparison of demographics by operative group.

	Total patients (shoulders)	Male sex, % (*n*)	Mean age, y (*n*)	Mean follow-up, m (*n*)
RSA	78 (78)	38.5 (65)	72.8 (65)	44.8 (50)
All TSA	396 (414)	58.5 (311)	63.7 (311)	54.1 (311)
TSA + ER	135 (135)	67.6 (74)	65.3 (74)	48.5 (74)
TSA + PAGC	83 (84)	71.1 (83)	65.4 (83)	46.7 (83)
TSA + BG	11∗ (11)	45.5∗ (11)	64.6∗ (11)	83.7∗ (11)
TSA + UGA	167 (184)	47.6 (143)	61.8 (143)	59.0 (143)

*RSA*, reverse shoulder arthroplasty; *TSA*, total shoulder arthroplasty; *ER*, eccentric reaming; *PAGC*, posteriorly augmented glenoid component; *BG*, bone grafting; *UGA*, unspecified glenoid approach.

∗ Data and patients from one study only.

### ROM and functional outcomes

Average active ROM improvements for patients undergoing TSA and RSA were 50.1 ± 8.5° and 64.7 ± 5.2° in flexion, 58.5 ± 10.3° and 68.9 ± not reported (NR)° in abduction, and 31.3 ± 5.7° and 29.0 ± 10.2° in external rotation, respectively. Average active ROM improvements for TSA + ER and TSA + PAGC patients were 58.1 ± 19.9° and 49.0 ± 4.2° in flexion, 45.0 ± NR° and 52.9 ± 8.1° in abduction, and 40.0 ± 12.3° and 31.0 ± 4.7° in external rotation, respectively. Complete ROM data are outlined by study in Table IV and compared as weighted averages in Table V. Regarding functional score outcomes, patients undergoing TSA and RSA showed average Constant score improvements of 38.8 ± 5.3 and 46.6 ± 3.1 points and ASES score improvements of 48.2 ± 1.0 and 49.2 ± 25.3 points, respectively. TSA + ER and TSA + PAGC subgroups demonstrated average Constant score improvements of 43.1 ± 4.8 and 36.1 ± 0.6 points and ASES score improvements of 48.9 ± 17.5 and 47.6 ± 1.0 points, respectively. Functional outcome scores as well as revision and complication rate data are outlined by the study in Table VI and compared as weighted averages in Table VII.

**Table IV:** Active range of motion outcomes for all included studies, organized by treatment group.

First author & year	Patients (shoulders), n	Flexion at final FU (SD or range), °	Δ Flexion (SD), °	Abduction at final FU (SD or range), °	Δ Abduction (SD), °	External rotation at final FU (SD or range), °	Δ External rotation, (SD), °
TSA + ER							
Alentorn-Geli et al, 2018^2^^,^∗	15 (15)	164.9 (±13.8)	77.6† (NR)	NR	NR	61.7 (±19.2)	52.1† (NR)
Egger et al, 2019^8^	15 (15)	165.4 (±16.2)	NR	NR	NR	56.1 (±17.9)	NR
Leschinger et al, 2017^23^	27 (27)	NR	NR	NR	NR	NR	NR
Magosch et al, 2020^28^	15 (15)	157.7 (±19.2)	38.5† (NR)	149.6 (±21.5)	45.0† (NR)	46.2 (±19.4)	27.9† (NR)
Orvets et al, 2018^36^	59 (59)	NR	NR	NR	NR	NR	NR
Pastor et al, 2015^38^	4 (4)	NR	NR	NR	NR	NR	NR
TSA + PAGC							
Grey et al, 2020^12^	46 (46)	151.0 (±16.0)	52.0 (±24.0)	143.0 (±25.0)	57.0 (±30.0)	58.0 (±18.0)	34.0 (±20.0)
Rice et al, 2008^39^	13 (14)	NR	NR	160.0 (120-180)	61.0† (NR)	56.0 (30-90)	21.0† (NR)
Wright et al, 2015^47^	24 (24)	142.0 (±14.0)	43.2 (±19.3)	133.4 (±20.4)	40.3 (±26.6)	46.3 (±23.8)	31.1 (±19.5)
TSA + BG							
Klika et al, 2014^21^	11 (11)	145.0 (±25.3)	NR	NR	NR	59.1 (±24.3)	NR
TSA + UGA							
Habermeyer et al, 2007^13^	24 (24)	NR	NR	NR	NR	NR	NR
Magosch et al, 2017^27^^,^∗	68 (68)	149.7 (±25.8)	54.9† (NR)	138.9 (±30.8)	68.5† (NR)	44.8 (±19.2)	29.1† (NR)
Walch et al, 2012^45^	75 (92)	143.3 (60-180)	44.9† (NR)	NR	NR	37.3 (0-60)	30.3† (NR)
RSA							
Alentorn-Geli et al, 2018^2^^,^∗	16 (16)	160.0 (±22.5)	73.2† (NR)	NR	NR	53.7 (±34.4)	43.7† (NR)
Collin et al, 2019^5^	15 (15)	147.0 (NR)	NR	NR	NR	18 (NR)	NR
Magosch et al, 2017^27^^,^∗	7 (7)	137.2 (±22.2)	60.1† (NR)	128.9 (±32.2)	68.9† (NR)	27.2 (±17.2)	11.2† (NR)
Mizuno et al, 2013^32^	27 (27)	152.0 (NR)	63.0† (NR)	NR	NR	27.0 (NR)	24.0† (NR)
Waterman et al, 2020^46^	13 (13)	NR	60.0 (±56.8)	NR	NR	NR	30.8 (±38)

Δ, change; *FU*, follow-up; *SD*, standard deviation; *TSA*, total shoulder arthroplasty; *ER*, eccentric reaming; *NR*, not reported; *PAGC*, posteriorly augmented glenoid component; *BG*, bone grafting; *UGA*, unspecified glenoid approach; *RSA*, reverse shoulder arthroplasty.

∗ Study includes both RSA and TSA B2 patients.

† Change calculated as the difference between final FU mean and preoperative mean.

**Table V:** Comparison of active range of motion outcomes by operative group.

	Total patients (shoulders)	Mean flexion at final FU, ° (*n*)	Mean Δflexion, ° (*n*)	Mean abduction at final FU, ° (*n*)	Mean Δabduction, ° (*n*)	Mean external rotation at final FU, ° (*n*)	Mean Δexternal rotation, ° (*n*)
RSA	78 (78)	151.2 (65)	64.7 (63)	128.9∗ (7)	68.9∗ (7)	31.5 (65)	29.0 (63)
All TSA	396 (414)	149.1 (286)	50.1 (260)	142.0 (167)	58.5 (167)	47.2 (300)	31.3 (274)
TSA + ER	135 (135)	162.7 (45)	58.1 (30)	149.6∗ (15)	45.0∗ (15)	54.7 (45)	40.0 (30)
TSA + PAGC	83 (84)	147.9 (70)	49.0 (70)	143.1 (84)	52.9 (84)	54.3 (84)	31.0 (84)
TSA + BG	11∗ (11)	145.0∗ (11)	NR (0)	NR (0)	NR (0)	59.1∗ (11)	NR (0)
TSA + UGA	167 (184)	146.0 (160)	49.2 (160)	138.9∗ (68)	68.5∗ (68)	40.5 (160)	29.8 (160)

Δ, change; *FU*, follow-up; *RSA*, reverse shoulder arthroplasty; *TSA*, total shoulder arthroplasty; *ER*, eccentric reaming; *PAGC*, posteriorly augmented glenoid component; *BG*, bone grafting; *NR*, not reported; *UGA*, unspecified glenoid approach.

∗ Data and patients from one study only.

**Table VI:** Outcome scores and complications for all included studies, organized by treatment group.

First author & year	Patients (shoulders), n	Constant score at final FU (SD or range), points	Δ Constant score (SD or range), points	ASES score at final FU (SD or range), points	ΔASES score (SD), points	Other outcome scores at final FU or Δ (SD or range), units specified by score	Complications, n (%)	Revisions, n (%)	Notes
TSA + ER									
Alentorn-Geli et al, 2018^2^^,^∗	15 (15)	NR	NR	91.0 (±6.7)	NR	SST score, points: 10.6 (NR)	4/15 (26.7)	0/15 (0.0)	4 shoulders were considered a radiographic failure: 2 demonstrated glenoid component loosening, 3 demonstrated failure of the posterior capsule plication (1 patient had both). Two additional shoulders developed progressive late cuff insufficiency with superior migration of the humeral component.
Egger et al, 2019^8^	15 (15)	NR	NR	NR	NR	- PSS-total, points: 86.3 (±14.0) - VAS score, mm: 1.4 (±1.5)	0/15 (0.0)	0/15 (0.0)	-
Leschinger et al, 2017^23^	27 (27)	NR	46.6 (-13 to 98)	NR	NR	NR	NR	0/27 (0.0)	Overall complication rate was 4.9%, B2’s not stratified.
Magosch et al, 2020^28^	15 (15)	77.9 (±12.8)	36.7† (NR)	NR	NR	- Relative Constant score, %: 104.5 (±23.4) - Δ Relative Constant score, %: 50.7† (NR)	NR	NR	Overall revision rate was 4.2%, B2’s not stratified. 1 revision to RSA was specified as a B2.
Orvets et al, 2018^36^	59 (59)	NR	NR	84.3 (±14)	48.9 (±17.5)	- SST score, points: 9.1 (±2.2) - VAS score, mm: 1.4 (±1.9)	1/59 (1.7)	1/59 (1.7)	1 shoulder sustained a rotator cuff tear and was converted to RSA.
Pastor et al, 2015^38^	4 (4)	58.4 (NR)	NR	NR	NR	NR	NR	NR	1 patient underwent arthroscopic capsular release for postoperative stiffness, not specified if B2. No clinical symptoms of loosening observed in any patients.
TSA + PAGC									
Grey et al, 2020^12^	46 (46)	78.4 (±10.9)	35.6 (±11.4)	89.7 (±15.0)	46.9 (±16.5)	- SPADI score, %: 11.6 (±19.2) - Δ SPADI score, %: 64.9 (±23.6) - SST score, points: 11.4 (±1.5) - Δ SST score, points: 5.6 (±2.7) - UCLA score, points: 32.2 (±4.2) - Δ UCLA score, points: 16.4 (±3.6)	NR	2/46 (4.3)	2 revisions for aseptic glenoid loosening reported, both specified as B2. 2 patients also reported as having axillary neurapraxia; however, glenoid morphology and/or overlap with revision patients not specified.
Rice et al, 2008^39^	13 (14)	NR	NR	NR	NR	- Neer Pain, points: 2.1 (NR) - Δ Neer Pain, points: 2.2 (NR) - Neer result rating: 5 excellent, 7 satisfactory, 2 unsatisfactory	4/14 (28.6)	0/14 (0.0)	4 shoulders demonstrated more than mild postoperative subluxation, 3 posterior and 1 anterior. 1 shoulder with posterior subluxation demonstrated shifting of the glenoid component and was considered radiographically loose.
Wright et al, 2015^47^	24 (24)	75.6 (±9.4)	36.9 (±14.2)	91.5 (±12.5)	48.9 (±21.0)	- SPADI score, %: 13.1 (±18.6) - Δ SPADI score, %: -64.0 (±28.9) - SST score, points: 11.0 (±1.5) - Δ SST score, points: 6.0 (±2.8) - UCLA score, points: 32.1 (±3.1) - Δ UCLA score, points: 16.9 (±6.4)	0/24 (0.0)	0/24 (0.0)	-
TSA + BG									
Klika et al, 2014^21^	11 (11)	NR	NR	NR	NR	Neer result rating: 7 excellent, 2 satisfactory, 2 unsatisfactory	2/11 (18.2)	2/11 (18.2)	2 shoulders revised for aseptic glenoid loosening and pain.
TSA + UGA									
Habermeyer et al, 2007^13^	24 (24)	65.4 (NR)	28.8† (NR)	NR	NR	- Relative Constant score, %: 89.1 (NR) - Δ Relative Constant score, %: 39.9† (NR)	NR	NR	Overall complication rate was 2.6%, B2’s not stratified.
Magosch et al, 2017^27^^,^∗	68 (68)	74.0 (±16.3)	45.6† (NR)	NR	NR	NR	NR	NR	11 TSA shoulders underwent revision surgery: 8 due to glenoid loosening, 1 due to atraumatic dislocation, 1 due to rotator cuff insufficiency, 1 due to a traumatic fracture. However, it was not specified which of these shoulders were a part of the rotator cuff intact subgroup.
Walch et al, 2012^45^	75 (92)	68.8 (25-95)	36.4† (NR)	NR	NR	NR	19/92 (20.7)	15/92 (16.3)	19 shoulders were deemed radiographically loose of which 11 had migration of the glenoid component. 15 shoulders underwent implant revision: 6 due to glenoid loosening, 5 due to posterior prosthesis dislocation. 4 more revisions occurred not involving the implant: 1 due to refractory capsulitis, 1 due to traumatic subscapularis tear, 1 due to persistent painful shoulder, and 1 due to impingement syndrome.
RSA									
Alentorn-Geli et al, 2018^2^^,^∗	16 (16)	NR	NR	80.0 (±14.3)	NR	SST score, points: 8.5 (NR)	0/16 (0.0)	0/16 (0.0)	2 shoulders were deemed to have “unsatisfactory” results due to a lack of external rotation. Notably these 2 patients had preoperative rotator cuff deficiency of 3+/5 and 4-/5 active external rotation, respectively.
Collin et al, 2019^5^	15 (15)	73.0 (NR)	NR	NR	NR	- Adjusted Constant score, %: 107 (NR) - SSV, %: 79 (NR)	NR	NR	The following are complications from the entire cohort, not specified if B2: 1 transient radial nerve palsy, 2 postop infections that required revisions.
Magosch et al, 2017^27^^,^∗	7 (7)	72.7 (±21.1)	52.6† (NR)	NR	NR	NR	NR	NR	7 RSA shoulders underwent revision surgery: 1 due to glenosphere loosening, 2 due to prosthetic instability, 2 due to a prosthesis infection, 1 due to periprosthetic fracture, 1 unaccounted for. However, it was not specified which of these shoulders were a part of the rotator cuff intact subgroup.
Mizuno et al, 2013^32^	27 (27)	76.0 (NR)	45.0† (NR)	NR	NR	NR	4/27 (14.8)	1/27 (3.7)	Complications included 1 glenoid loosening, 1 transient axillary nerve palsy, 1 transient ulnar nerve palsy, 1 permanent ulnar nerve palsy. The 1 revision was for glenoid loosening and was to hemiarthroplasty.
Waterman et al, 2020^46^	13 (13)	NR	NR	NR	49.2	- Δ SANE score, points: 71.8 (±44.9) - Δ SST score, points: 7.6 (±3.1) - Δ VAS score, mm: 4.4 (±3.0)	NR	NR	Authors did not report preoperative or postoperative clinical results aside from change in clinical scores.

Δ, change; *ASES*, American Shoulder and Elbow Surgeons; *FU*, follow-up; *SD*, standard deviation; *TSA*, total shoulder arthroplasty; *ER*, eccentric reaming; *NR*, not reported; *SST*, simple shoulder test; *PSS*, Penn Shoulder Score; *VAS*, Visual Analog Scale; *RSA*, reverse shoulder arthroplasty; *PAGC*, posteriorly augmented glenoid component; *SPADI*, Shoulder Pain and Disability Index; *UCLA*, University of California, Los Angeles; *BG*, bone grafting; *UGA*, unspecified glenoid approach; *SSV*, subjective shoulder value; *SANE*, Single Assessment Numeric Evaluation.

∗ Study includes both RSA and TSA B2 patients.

† Change calculated as the difference between final FU mean and preoperative mean.

**Table VII:** Comparison of outcome scores and complication rates by operative group.

	Total patients (shoulders)	Mean Constant score at final FU, points (*n*)	Mean ΔConstant score, points (*n*)	Mean ASES score at final FU, points (*n*)	Mean ΔASES score, points (*n*)	Complications, all, n (%)	Mean revisions, n (%)
RSA	78 (78)	74.6 (49)	46.6 (34)	80∗ (16)	49.2∗ (13)	4/43 (9.3)	1/43 (2.3)
All TSA	396 (414)	72.4 (273)	38.8 (296)	87.9 (144)	48.2 (129)	30/230 (13.0)	20/303 (6.6)
TSA + ER	135 (135)	73.8 (19)	43.1 (42)	85.7 (74)	48.9∗ (59)	5/89 (5.6)	1/116 (0.9)
TSA + PAGC	83 (84)	77.4 (70)	36.1 (70)	90.3 (70)	47.6 (70)	4/38 (10.5)	2/84 (2.4)
TSA + BG	11∗ (11)	NR (0)	NR (0)	NR (0)	NR (0)	2/11∗ (18.2)	2/11∗ (18.2)
TSA + UGA	167 (184)	70.3 (184)	38.8 (184)	NR (0)	NR (0)	19/92∗ (20.7)	15/92∗ (16.3)

Δ, change; *ASES*, American Shoulder and Elbow Surgeons; *FU*, follow-up; *RSA*, reverse shoulder arthroplasty; *TSA*, total shoulder arthroplasty; *ER*, eccentric reaming; *PAGC*, posteriorly augmented glenoid component; *BG*, bone grafting; *NR*, not reported; *UGA*, unspecified glenoid approach.

∗ Data and patients from one study only.

### Complication and revision rates

**Figure 2:**
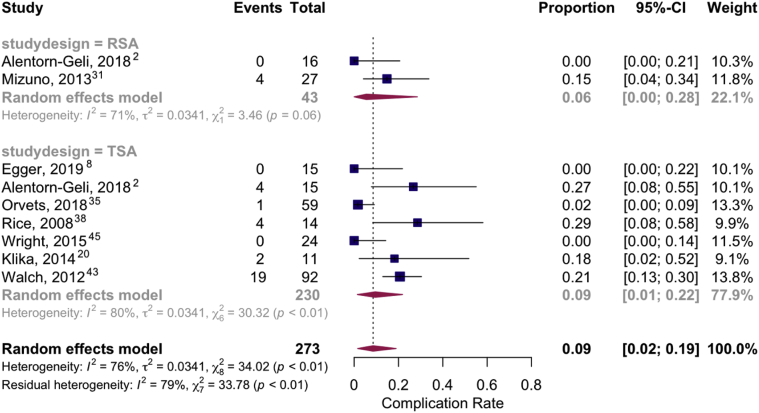
Forest plot of pooled complication rates: RSA vs. TSA.

**Figure 3:**
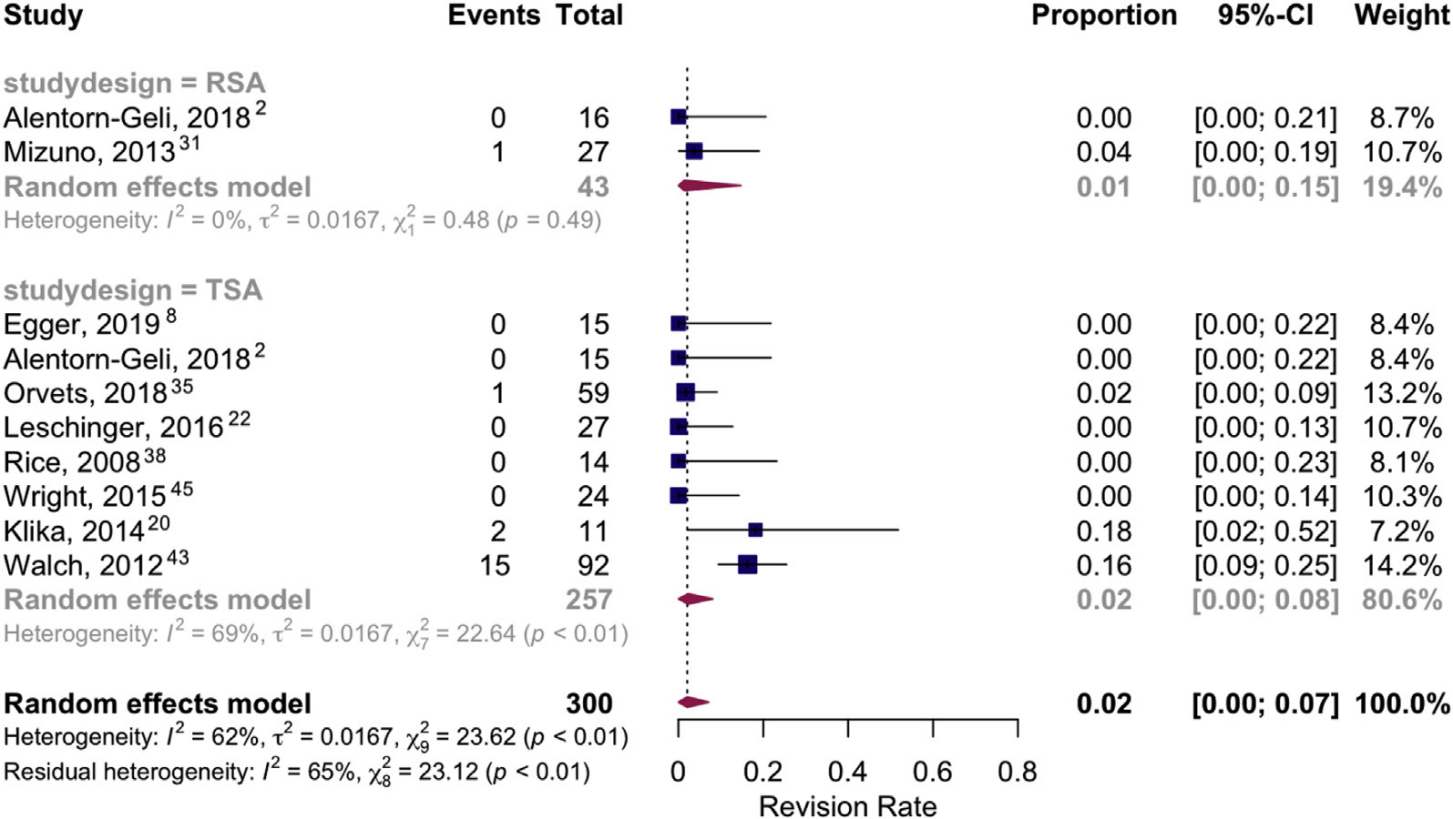
Forest plot of pooled revision rates: RSA vs. TSA.

Overall pooled complication and revision rates after RSA and TSA were evaluated in a meta-analysis of proportions model. The pooled complication rate among the 2 studies that reported on RSA was 6% (95% CI, 0%-28%) with substantial heterogeneity (I^2^ = 71%) compared to the 7 studies that reported on TSA with a rate of 9% (95% CI, 1%-22%) with substantial heterogeneity (I^2^ = 80%). The pooled revision rate among the 2 studies that reported on RSA was 1% (95% CI, 0%-15%) with no heterogeneity (I^2^ = 0%) compared to TSA that had a reoperation rate of 2% (95% CI = 0%-8%) with substantial heterogeneity (I^2^ = 69%). The forest plots of pooled complication and revision rates are presented as Figures 2 and 3, respectively.

For the TSA + ER subgroup, the complication rate was 5.6% (5/89 shoulders), and the revision rate was 0.9% (1/116 shoulders). Two shoulders had GCL, 3 had failure of the posterior capsule plication procedure (1 had both), and 1 had a rotator cuff tear that underwent conversion to RSA. For the TSA + PAGC subgroup, the complication rate was 10.5% (4/38 shoulders), and the revision rate was 2.4% (2/84 shoulders). The majority of these complications were glenohumeral subluxation (3 posterior, 1 anterior), of which 1 also demonstrated GCL. The 2 revisions were for GCL. For the TSA + BG subgroup, the complication and revision rates were both 18.2% (2/11 shoulders). These 2 shoulders were revised for GCL. For the TSA + UGA subgroup, the complication rate was 20.7% (19/92 shoulders), all from radiographic GCL. The revision rate was 16.3% (15/92 shoulders), of which the majority were for GCL (6 shoulders) and posterior shoulder dislocations (5 shoulders). Four RSA shoulders were noted for complications. Of these, 3 were nerve palsies (1 axillary, 2 ulnar) and 1 was GCL which was revised to hemiarthroplasty.

## Discussion

The purpose of this systematic review is to compare the surgical, radiographic, and functional outcomes of patients with symptomatic glenohumeral joint osteoarthritis and a Walch B2 glenoid without significant rotator cuff pathology managed with either anatomic TSA vs. RSA. We hypothesized that a systematic review of the literature would demonstrate patients with B2 glenoid morphology having superior outcomes and a lower complication and revision rate after RSA than TSA. In regard to ROM and clinical score outcomes, the results of our review suggest largely similar outcomes between RSA, all TSA, and the TSA subgroups in the setting of B2 glenoid morphology. Regarding complications, similar rates were observed between the TSA + ER (5.6%) and RSA (6%, pooled rate from the meta-analysis) study groups, while more significant rates were observed in the TSA + PAGC (10.5%), TSA + BG (18.2%), and TSA + UGA (20.7%) subgroups. Revision rates of <3% were also demonstrated for all study groups with the exception of the TSA + BG and TSA + UGC subgroups which demonstrated much higher rates of 18.2% and 16.3%, respectively. Overall, these results suggest similar outcomes between the traditional TSA + ER approach and RSA for B2 glenoids, with newer approaches of TSA + PAGC and TSA + BG potentially leading to more complications and the need for revision. It is important to note that the 92 shoulders from the review by Walch et al^45^ on ER for B2 glenoids were not included in the TSA + ER subgroup and instead contribute to the TSA + UGA subgroup; however, the results described by that study, particularly the high complication and revision rates, should also be considered when interpreting the outcomes of the TSA + ER subgroup from our analysis.

Neer et al^34^ recognized that posterior glenoid erosion and static posterior humeral head subluxation was a common wear pattern in symptomatic glenohumeral joint osteoarthritis. Nearly 20 years later, Walch et al^44^ described the B2 glenoid as one with static posterior humeral head subluxation and a “posterior cupula,” giving the glenoid a biconcave morphology. Since then, the challenge of managing the unique B2 glenoid morphology has been an area of great interest for shoulder surgeons. Multiple techniques including TSA in combination with ER, PAGC, and BG as well as RSA have been described to address this deformity.

### TSA with eccentric reaming (TSA + ER)

In the ER technique, the anterior glenoid is reamed eccentrically to eliminate the biconcavity, correct retroversion, and provide a stable plane for the glenoid component of anatomic TSA. Generally, surgeons aim to correct glenoid retroversion to ≤5-10° to neutral version. This is in an effort to optimize the glenohumeral contact point, preventing the implant stress that can arise from retroverted implants as has been described in several biomechanical studies.^9^^,^^40^ Work by Ho et al^16^ supported this clinically in their series of 66 shoulders, demonstrating increased osteolysis rates around the center glenoid peg and loosening when component retroversion was ≥15°. It is important to consider, however, that ER in cases of severe deformity involves significant glenoid bone stock removal possibly contributing to GCL and excessive joint line medialization that can disrupt rotator cuff tensioning.^1^^,^^4^^,^^26^ Our review demonstrated superior outcomes in mean flexion (162.7 ± 3.6°) and abduction (149.6 ± 21.5°) at the final FU as well as external rotation improvement (40.0 ± 12.3°) in patients in whom this technique was used. A complication rate of 5.6% and revision rate of 0.9% were reported when the version was corrected to ≤5-10° of retroversion to neutral.

In 2012, Walch et al^45^ described the results of 92 B2 glenoids treated with TSA + ER, most notably reporting a 20.6% GCL rate and a 16.3% revision rate in this cohort. In cases of intermediate glenoid retroversion >30°, the rate of complications reached 62%. This is in contrast to a similar study by Orvets et al^36^ who found no instances of GCL in their cohort of 59 B2 glenoids treated with TSA + ER. In addition, there was no difference in the rate of glenoid radiolucent lines between shoulders with a preoperative glenoid retroversion of ≤20° (27.8%) vs. >20° (22.7%, *P* = .670). However, the authors acknowledged that their cohort did not include patients with >30° of glenoid retroversion. Furthermore, the mean radiographic FU was 32 months vs. 77 months in the study by Walch et al.^45^ Additional studies by Leschinger et al^23^ and Egger et al^8^ support TSA + ER to be an effective option for B2 glenoids regarding functional outcomes, and neither described any revisions in B2 patients. However, neither study indicated the degree of preoperative retroversion in their cohorts. Therefore, overall, the studies by Orvets et al^36^ and Walch et al^45^ provide the clearest data which may indicate that ER is not appropriate for glenoid retroversion >30° but is a reasonable option for those with retroversion <30°. It is also important to note that as many of the studies do not have long-term FU, it is difficult to assess the true rate of GCL.

### TSA with bone grafting (TSA + BG)

In the glenoid BG technique, asymmetric autograft (typically from the humeral head) is fixated with 2 or 3 independent screws before glenoid component implantation to correct retroversion. In our analysis, the TSA + BG study group consisted entirely of 11 B2 glenoids reported on by Klika et al.^21^ At a mean FU of 7.6 years, 2 patients (18.2%) required revisions for GCL. In addition, on final radiographs, 4 patients (16%) demonstrated glenoid lucent lines >1.5 mm, 5 (25%) demonstrated a shift in glenoid component position, 5 (25%) demonstrated graft resorption or lack of incorporation, and 7 (28%) revealed the glenoid component to be at risk for failure. As mentioned previously, Walch et al^45^ also reported on the use of BG in 7 of their 92 B2 glenoids of which 2 (28.6%) demonstrated graft collapse and 3 (42.9%) presented with posterior dislocation of which 2 (28.6%) underwent revision with graft removal. In summary, these studies suggest the use of posterior BG to correct glenoid retroversion should be approached with caution and that high rates of GCL, graft collapse, and dislocation may be encountered.

### TSA with a posteriorly augmented glenoid component (TSA + PAGC)

PAGCs were developed to allow for glenoid retroversion correction while avoiding glenoid subchondral bone loss and excessive joint medialization. Early studies demonstrated good results and low revision rates as compared to TSA + BG and TSA + ER.^26^ Our analysis demonstrated good to excellent outcomes for TSA + PAGCs in mean Constant score (77.4 ± 1.3 points) and ASES score (90.3 ± 0.9 points) at the final FU. Interestingly, the TSA + PAGC subgroup also demonstrated the lowest mean improvements in Constant score (36.1 ± 0.6 points) and ASES score (47.6 ± 1.0 points) of any group. The revision rate for this group was only 2.4%; however, the complication rate was higher at 10.5% most commonly because of posterior glenohumeral subluxation.

Rice et al^39^ first reported the outcomes of 14 B2 glenoids treated with a 4° PAGC in which, at a mean FU duration of 5 years, all but 2 patients (85.7%) demonstrated excellent or satisfactory outcomes on the Neer Result Rating. Notably, 4 patients (28.6%) demonstrated humeral head subluxation (3 posterior, 1 anterior), 1 patient (7.1%) demonstrated grade 5 radiolucent lines, and 1 patient (7.1%) demonstrated radiographic GCL although no revisions were performed. In a separate investigation by Grey et al,^12^ significant improvement in multiple functional scores and ROM outcomes was demonstrated in 46 B2 glenoids that received an 8° PAGC. Of these, 2 shoulders (4.3%) demonstrated GCL and underwent revision. Finally, a recent study by Wright et al^47^ demonstrated that equivalent patient-reported and objective outcomes can be expected in B2 glenoids treated with PAGC (8° or 16°) in comparison to a nonaugmented component (NAGC) albeit in shoulders of unspecified glenoid morphology. The study did mention, however, that radiographic results favored the NAGC group, with 12 of 20 PAGC patients (60%) demonstrating a radiolucent line vs. 5 of 15 NAGC patients (33.3%).

### RSA with RCI for B2 glenoid

RSA has recently been advocated for as a treatment option for B2 glenoids in RCI shoulders. In theory, the semi-constrained design addresses the problems of recurrent posterior humeral head subluxation and posterior instability while the robust glenoid base plate fixation decreases the likelihood of GCL. However, surgeons may end up encountering a different set of complications unique to RSA.^37^ In our analysis, we found B2 glenoids treated with RSA to have superior mean improvements in flexion (64.7 ± 5.2°) and abduction (68.9 ± NR°) but inferior mean improvement in external rotation (29.0 ± 10.2°). Mean abduction (128.9 ± 32.2°) and external rotation (31.5 ± 13.3°) at the final FU were also inferior to those of all TSA subgroups. Results from our meta-analysis also demonstrated a complication rate of 6% and revision rate of 1% for B2 glenoids treated with RSA.

In a recent study by Collin et al,^5^ 15 RCI B2 glenoids were treated with RSA and demonstrated mean active flexion of 147°, external rotation of 18°, and Constant score of 73 points at the final FU. Mizuno et al^32^ reported similar results in their cohort of 27 RCI B2 glenoids in which patients achieved a mean improvement in active flexion of 63°, active external rotation of 24°, and Constant score of 45 points. Mizuno et al^32^ also commented on complications including 1 case (3.7%) of GCL and 3 cases (11.1%) of nerve palsy (1 axillary, 2 ulnar). There was 1 patient (3.7%) who underwent revision to hemiarthroplasty. Notably 10 B2 glenoids (37.0%) in this cohort received additional BG. Collin et al^5^ also described the use of BG in their entire study cohort; however, it is not specified how many were of B2 morphology. Although limited by small cohorts and limited outcomes reporting, these studies suggest that RSA may be a viable treatment choice for RCI B2 glenoids.

### Studies comparing TSA to RSA for B2 glenoids

Recently, studies have aimed to directly compare TSA to RSA for patients with RCI B2 glenoids. Recent work by Alentorn-Geli et al^2^ demonstrated that TSA + ER and RSA shoulders have comparable mean active ROM improvements. In regard to outcome scores, the TSA + ER group demonstrated a superior final mean Simple Shoulder Test Score (10.6 vs. 8.5 for RSA) and was trending toward superiority in final mean ASES score (91.2 ± 6.7 vs. 80.3 ± 14.3 for RSA). Radiographically, however, 9 patients (60%) undergoing TSA experienced some degree of radiolucent lines with 4 patients (26.7%) deemed as radiographic failures. In addition, 2 patients (13.3%) demonstrated GCL, 3 (20%) had failure of the posterior capsule plication, and 2 (13.3%) developed progressive late rotator cuff insufficiency although no revisions were performed. This is in comparison to no radiolucent lines or GCL in the RSA cohort. Overall, this study suggests similar pain relief and improvement in ROM between approaches; however, TSA + ER may offer superior functional outcomes although with a greater risk of GCL and soft-tissue failure. In a similar study by Magosch et al,^27^ a comparable Constant score and active ROM improvement were demonstrated between RCI TSA and RSA study groups, with the only appreciable difference being in external rotation improvement (29.1° for patients undergoing TSA vs. 11.2° for patients undergoing RSA). Unfortunately, revision rates were NR for RCI shoulders specifically; however, the RSA group experienced a higher revision rate of 21.2% (7/33 shoulders) vs. 12.8% (11/86 shoulders) for the TSA group. In comparison to these studies, results from our analysis demonstrated >10° differences in mean flexion and abduction improvements favoring RSA, but conversely >10° differences in mean abduction and external rotation at the final FU favoring TSA. Mean Constant score improvement was also 7.8 points higher in patients undergoing RSA, but the mean ASES score at the final FU was 7.9 points higher in patients undergoing TSA. Results from the meta-analysis demonstrated a higher complication rate of 9% in patients undergoing TSA than the 6% in patients undergoing RSA but similar revision rates of 2% in TSA and 1% in RSA.

Limitations of this study include that this review is dependent on the data reported by each study and its quality. In studies that included only patients with B2 glenoids, data collection was limited to which outcome variables the investigators chose to collect and report. In instances where multiple Walch glenoid types were studied, data collection was further limited to the degree to which the investigators stratified data based on morphology. In the articles included in this review, some form of outcome data specifically pertained to patients with B2 glenoids, although for many of these articles, demographics data were not stratified.

Many articles were excluded from this review for not specifying the patterns of glenoid erosion or, if specified, not stratifying outcomes for the B2 subgroup. In addition, 3 TSA studies included in our review did not specify the glenoid correction technique limiting our subgroup analysis. Most articles also did not specify mean glenoid retroversion or degree of humeral head subluxation, making it difficult to draw conclusions and form recommendations regarding treatment choice for specific ranges of retroversion. This review is also limited by the small sample size in the RSA group, due to a lack of literature on outcomes after RSA for RCI B2 glenoids. All studies except one included in this review were classified as level III or IV evidence. Finally, outcomes after a procedure are likely affected not only by a surgeon’s overall experience and skill but also by their experience and skill in a specific procedure.

Strengths of the study are that this is the most comprehensive review of the literature regarding outcomes after TSA and RSA in patients with B2 glenoids without significant rotator cuff pathology. We used well-defined search criteria to identify articles with pertinent, applicable data. We included multiple objective and patient-reported outcome measures to compare the results of various surgical treatments. In addition, we took care to stratify results based on RSA vs. TSA, but we also further stratified the TSA group based on the correction technique used.

## Conclusion

This systematic review found comparable outcomes following RSA, TSA + ER, and TSA + PAGC for the treatment of patients with Walch B2 glenoids. Similar mid-term outcomes can be expected in regard to ROM improvement, functional score improvement, and revision rate if TSA + ER, TSA + PAGC, or RSA is chosen. A similar complication rate between TSA + ER and RSA can also be expected; however, the use of TSA + PAGC may increase the risk of glenohumeral subluxation. Overall, the results are limited by a lack of specificity on the severity of retroversion in studies. Treatment choice, therefore, should be made through a shared decision-making process and based on surgeon preference, patient age, activity level, and potentially by selecting the best plan with the assistance of newer preoperative planning tools. There remains the need for a randomized controlled trial with a large sample size and long-term FU to determine if any difference in outcome can be expected between these treatment options. Future studies are also indicated to determine the impact of initial glenoid version correction on outcomes in the B2 glenoid, particularly how much is absolutely necessary for proper implant stability as well as more detailed risks associated with correction techniques. Finally, future investigations of patients undergoing RSA and TSA should aim to report preoperative Walch classification using standardized preoperative imaging (CT scans) for measurement, subjective and objective data, and stratify outcomes based on accurate classification of the Walch morphology to allow for more accurate comparison across studies.

## Disclaimers

Funding: No funding was disclosed by the authors.

Conflicts of interest: The authors, their immediate family, and any research foundation with which they are affiliated have not received any financial payments or other benefits from any commercial entity related to the subject of this article.
